# Preventive and Therapeutic Effects of Astaxanthin on Depressive-Like Behaviors in High-Fat Diet and Streptozotocin-Treated Rats

**DOI:** 10.3389/fphar.2019.01621

**Published:** 2020-01-30

**Authors:** Yuting Ke, Shizhong Bu, Hong Ma, Lei Gao, Yujia Cai, Yisheng Zhang, Wenhua Zhou

**Affiliations:** ^1^ Laboratory of Behavioral Neuroscience, Ningbo Addiction Research and Treatment Center, School of Medicine, Ningbo University, Ningbo, China; ^2^ School of Pharmacy, Shanghai Jiao Tong University, Shanghai, China; ^3^ Department of Neurology, Massachusetts General Hospital and Harvard Medical School, Charlestown, MA, United States; ^4^ Center of Diabetic Research, School of Medicine, Ningbo University, Ningbo, China; ^5^ Lihuili Eastern Hospital, School of Medicine, Ningbo University, Ningbo, China

**Keywords:** astaxanthin, diabetes mellitus type 2, depression, hyperlipidemia, brain-derived neurotrophic factor

## Abstract

The comorbidity of diabetes and depression has a negative impact on both lifestyle and quality of life. Astaxanthin (AST) has been demonstrated to improve glucose metabolism and has antidepressant-like effects, but it is not clear whether AST has potential for preventing depression in diabetes. The aim of this study is to observe the preventive and therapeutic effects of AST on glucose metabolism or depressive-like behaviors in a diabetic rat model produced by feeding with a high-fat diet for 10 weeks followed by injection of 25 mg/kg streptozotocin (STZ). Preventive treatment with AST at doses of 7.5, 15, and 25 mg/kg/day was given by intragastric gavage 4 weeks before STZ injection. Preventive plus therapeutic treatment also involved therapeutic AST treatments for 6 more weeks after STZ injection, whereas therapeutic-only treatment involved only the 6-week post-STZ treatment. Depressive-like behaviors were evaluated at the end of the treatment by using open field, locomotor activity, elevated plus maze, and forced swimming tests. Preventive and therapeutic treatment with AST both reduced the level of fasting glucose, improved glucose tolerance, and decreased total TCh and TG in diabetic rats. Preventive or preventative plus therapeutic treatment with AST decreased the immobility time and increased the time spent in the open arms of an elevated plus maze and locomotor activity in diabetic rats. However, therapeutic treatment with AST alone failed to affect the depressive-like behaviors. Preventive or preventative plus therapeutic treatment with AST at doses of 15 or 25 mg/kg significantly increased the expression of pERK, pAKT, pCREB, and BDNF in the prefrontal cortex (PFC) in diabetic rats. In contrast, therapeutic treatment with 25 mg/kg AST alone increased the expression of pERK in the PFC. This study indicates that AST may be used as a preventive or therapeutic approach for co-morbidity of diabetes and depression.

## Introduction

Diabetes mellitus (DM) and depression are common chronic diseases that threaten global health. The prevalence of depression in patients with diabetes has been widely reported (Katon, 2008). Rates of depression are significantly enhanced in diabetic patients: near 20-30% of diabetes patients suffered from clinically relevant depressive disorders (Fiore et al., 2015), and the risk of depression remains elevated over time in some type 2 diabetes patients (Whitworth et al., 2017). The metabolic dysfunction of obesity contributes to the development of depression (Carmo-Silva and Cavadas, 2017), and the interaction between depressive symptoms and metabolic dysfunction may be a risk factor for type 2 diabetes (Schmitz et al., 2016). Alteration in monoamines (serotonin and noradrenaline), increasing cortisol levels and decreasing levels of brain-derived neurotrophic factor (BDNF), could explain the association between depression and diabetes in some of the abnormalities documented in diabetic patients and animal models (Oladeji and Gureje, 2013; Lenart et al., 2016). Though high rates of depression are observed in patients with DM, the pathogenesis of co-morbid depression and diabetes is not certain.

Astaxanthin (AST) is a carotenoid pigment with multiple pharmacological properties (Wu et al., 2015). The beneficial effects of AST are associated with its anti-oxidative, anti-inflammatory, and anti-apoptotic properties (Grimmig et al., 2017). Several studies have demonstrated that AST potentially plays a neuroprotective role in neurological disorders, such as brain ischemic or traumatic injury and subarachnoid bleeding (Zhang et al., 2016; Ji et al., 2017; Masoudi et al., 2017). AST displays a protective effect for patients who are vulnerable to ischemic events (Pan et al., 2017), reduces isoflurane-induced neuron apoptosis (Wang et al., 2016), and prevents neurotoxicity caused by excessive alcohol consumption (Yan et al., 2016). Furthermore, AST is able to improve cognitive functions by protecting neurons against inflammation injury in vascular dementia (Hussein et al., 2005) and to suppress memory impairment caused by repeated cerebral ischemia and reperfusion (Xu et al., 2015) or by free radical-promoted neurodegenerative processes (Barros et al., 2014).

Moreover, AST not only ameliorates diabetic endothelial dysfunction (Zhao et al., 2011) but also attenuates hyperglycemia and inhibits the formation of advanced glycation end-products (Park et al., 2015). Accumulating evidence shows that AST provides neuroprotection against diabetes-induced sickness behavior through inhibiting inflammation (Ying et al., 2015), attenuating oxidative stress (Li et al., 2016), and causing the expression of caspase-3/9 in the cerebral cortex and hippocampus (Xu et al., 2015). A few studies demonstrate that AST decreases depressive-like behaviors stimulated by LPS or in diabetic mice *via* its potent anti-inflammatory effects (Zhou et al., 2015; Jiang et al., 2016; Zhou et al., 2017), and the evidence also shows that the serotonergic system may be involved in the antidepressant-like effect of AST (Jiang et al., 2016). Although AST improves both depression and diabetes, the underlying mechanism is unclear.

We hypothesized that chronic supplementation with AST may play a beneficial role in depression and glucose metabolism in the type 2 diabetic rat model. In this study, we observed the preventive or therapeutic effects of chronic treatment with AST on glucose metabolism or depressive-like behaviors in a diabetic rat model developed by feeding the rats with a high-fat diet (HFD) followed by a low dose of streptozotocin (STZ), which induces stable and typical characteristics of type 2 diabetes such as hyperglycemia, lipid disorder, and insulin resistance (Srinivasan et al., 2005). We then analyzed the expression of BDNF, phosphorylated extracellularsignal-regulated kinase (pERK), cyclic-AMP response element-binding protein (pCREB), and protein kinase B (pAKT) in the prefrontal cortex (PFC) in AST-treated rats.

## Materials and Methods

### Animals

Male Sprague-Dawley rats (300-350 g) purchased from the Zhejiang Experimental Animal Center were used. All animals were housed in a temperature-controlled (22-24°C) and relative humidity-controlled (50-60%) room with a 12-h light/dark cycle (lights on at 07:00, off at 19:00). All rats had free access to food and water. The experimental procedures were approved by the Institutional Animal Care and Use Committee of Ningbo University, and all animal experiments were performed according to the National Institutes of Health (NIH) Guide for the Care and Use of Laboratory Animals.

### Drugs and Materials

AST (purity ≥98%, 1 g/ml, and diluted with olive oil for different doses) was purchased from Ningbo Red Dragon Biotechnology Co., Ltd (Zhejiang, China). STZ was purchased from Sigma-Aldrich (St. Louis, MO, USA). HFD food was purchased from Shanghai Laboratory Animal Co., Ltd. (Shanghai, China).

### Experimental Design

**Figure 1:**
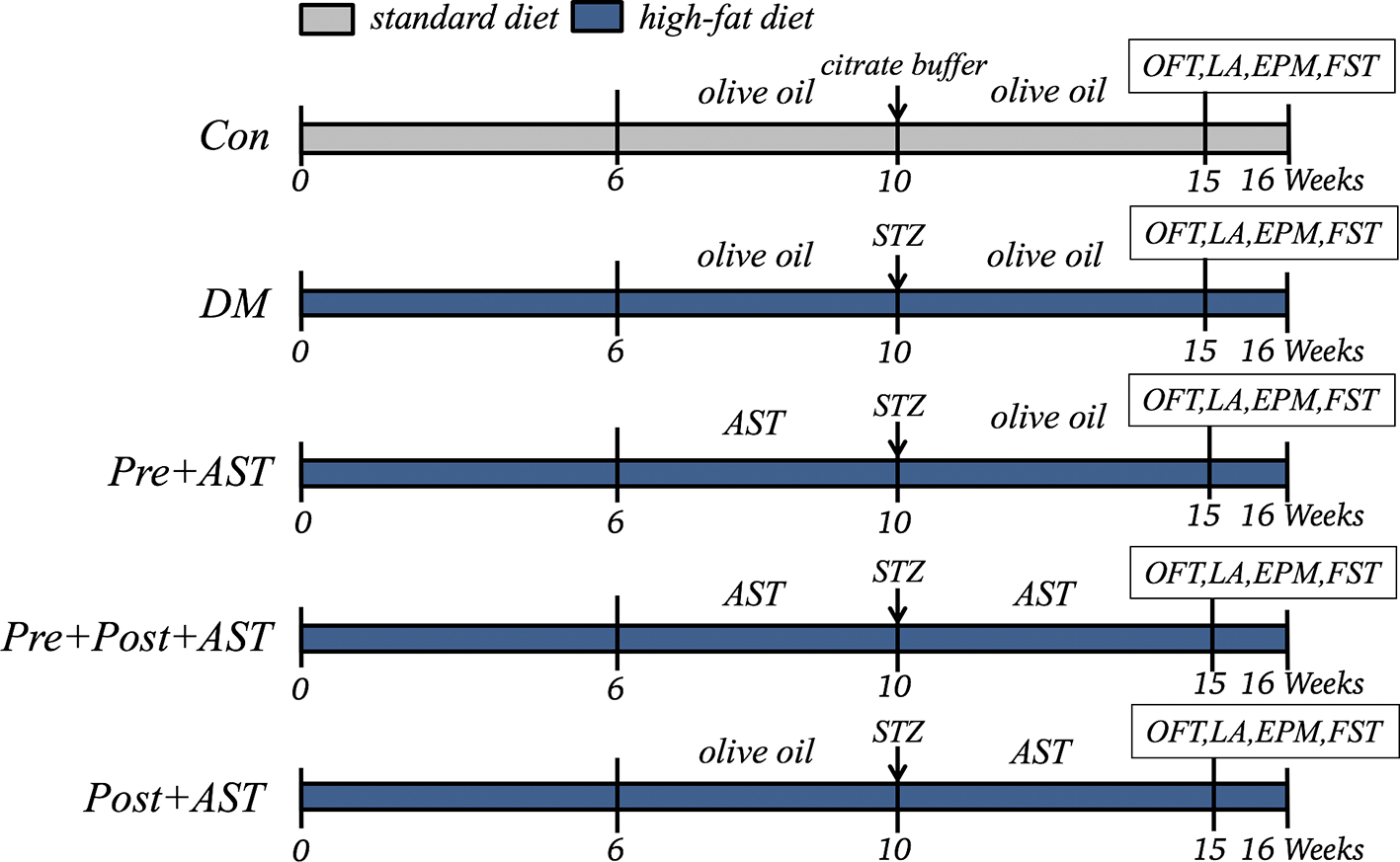
Experimental Schedules. Con, control; DM, diabetes mellitus; Pre+AST, preventive treatment with AST; Pre+Post+AST, preventive plus therapeutic treatment with AST; Post+AST, therapeutic treatment with AST; AST, astaxanthin; STZ, streptozocin; OFT, open field test; LA, locomotor activity; EPM, elevated plus-maze; FST, forced swimming test.

After an adaptive period of one week, rats were randomly divided into two matched groups: nondiabetic control and diabetes. The control group (Con, n = 6) was fed a standard diet. Other diabetes groups were fed an HFD. Diabetic rats were randomly assigned to DM, Pre+AST (7.5, 15, 25 mg/kg), Pre+Post+AST (7.5, 15, 25 mg/kg) and Post+AST (25 mg/kg) groups (n = 6 in each group). After 10 weeks of HFD feeding, a single dose of 25 mg/kg STZ dissolved in citrate buffer (pH 4.4, 0.1 M) was injected intraperitoneally (i.p.) into the rats in order to induce diabetes after fasting for 12 h. Age-matched control rats also received an equal volume of citrate buffer. The diabetic model was verified 72 h after STZ injection using a glucometer, and blood samples were collected through the tail vein. The rats were considered diabetic and kept in the study when non-fasting plasma glucose ≥16.7 mmol/L (Srinivasan et al., 2005). The experiments on AST intervention in diabetes groups were divided into preventive, preventive plus therapeutic, and therapeutic treatment-only groups. In the preventive treatment, the Pre+AST group of rats orally received AST at doses of 7.5 mg/kg/day (1.5 mg/ml diluted in olive oil), 15 mg/kg/day (3 mg/ml diluted in olive oil), or 25 mg/kg/day (5 mg/ml diluted in olive oil) for 4 weeks before STZ injection followed by 6 weeks of treatment with olive oil after STZ injection. In the preventive plus therapeutic AST treatment, the Pre+Post+AST group of rats orally received AST at doses of 7.5, 15, or 25 mg/kg/day for 4 weeks before STZ injection followed by continued administration for 6 weeks after STZ injection. The rats in the Post+AST group orally received olive oil for 4 weeks before STZ injection, followed by receiving AST at a dose of 25 mg/kg/day for 6 weeks after STZ injection to observe the therapeutic effect of AST. The AST was orally administrated by using a sonde by gavage. The Con and DM groups were orally treated daily with olive oil in the same volume as was used for AST. In the 15^th^ week, after measuring the blood glucose levels, all animals were assessed *via* behavioral tests at 2 h after the olive oil or AST treatment to evaluate their depressive-like behaviors (the experimental process is shown in **Figure 1**). Testing was performed between 4 p.m. and 11 p.m, and all rats were transported to the testing room to acclimatize 1 h before tests. Each test was separated by at least 3 days to prevent interference with the others. In all of the behavioral experiments, rats were randomized, and experimenters were blinded to animal groups during the behavioral tests and data analysis.

### Oral Glucose Tolerance Test (OGTT) and Lipid Parameters

The oral glucose tolerance test (OGTT) was carried out one week (11th week) and five weeks (15th week) after STZ injection. Following a 12-h fast, the rats were administered a glucose solution, which was given at a dose of 2 g glucose/kg body weight (500g/L) at 8:00 a.m. The concentration of blood glucose from the tail bleeding was analyzed at 0, 30, 60, and 120 min after glucose loading by One Touch Glucose Monitor (Johnson & Johnson, Shanghai, China). At the end of the study, all rats were fasted for 12 h, and blood samples were collected when the rats were sacrificed. The blood samples were immediately centrifuged at 3000 rpm for 10 min (4°C) to obtain plasma. Total cholesterol (TCh), triglycerides (TG), high-density lipoprotein cholesterol (HDL-C) and low-density lipoprotein cholesterol (LDL-C) levels were analyzed immediately by using a MODULAR P800 Automated Biochemist Analyzer (Roche, Basel, Switzerland).

### Open Field Test

The open field test (OFT) was performed as a standard procedure for measuring spontaneous activity and anxiety-like and exploratory behaviors in rodents (Blokland et al., 2002). Briefly, rats were put individually in the center of the box, which consists of a white Plexiglas box (100×100×40 cm) with a bottom divided into four identical squares on the floor of the arena, and were allowed to explore the field freely for 5 min. The test room was dimly illuminated. The number of line crossings and rearings was recorded automatically over 5 min by the video tracking system (SMART video tracking system, MoblieDatum, China). All behaviors were recorded automatically using a video camera located above the arena. After each test, the apparatus was cleaned with 70% ethanol to remove scent clues.

### Elevated Plus Maze Test

The elevated plus maze (EMP) test is widely used to study anxiety responses (Walf and Frye, 2007). The standard elevated plus maze apparatus consists of two opposing open arms (50 × 10 cm) and two opposing closed arms (50 × 10 × 40 cm) connected by a central platform (10 × 10 cm) and elevated 70 cm above the floor. During a test session, each rat was put gently on the center square, directed facing an open arm, and then allowed to explore the maze freely for 5 min in a sound-attenuated room. Their behavior was record by a video camera located over the arena using a video tracking system (SMART video tracking system, MoblieDatum, China). The test maze was wiped with a 70% ethanol solution after each trial. The time spent in the open arms and numbers of entries into the open or closed arms were recorded, and either the percentage of time spent or percentage of entries into the open arms was calculated based on the total time or the total number of entries; these values have been used as anxiety indices.

### Forced Swimming Test

The forced swimming test has previously been used to assess the antidepressant-like effects of drugs on rats (Porsolt et al., 1978). Rats were introduced into a clear plastic cylinder (height 40 cm, diameter 30 cm) filled with fresh water to a depth of 25 cm at 25°C, as described previously (Brocardo et al., 2007). The test comprised two sessions. In the first session, rats were individually allowed to swim for 15 min. Approximately 24 hours after the first session, rats were reintroduced into the same cylinder, and their 5 min swim session was recorded on video recorder (SONY, Tokyo, Japan). After each test, rats were dried with paper towels and placed in a separate cage, heated by a 300W lamp until the animal was dry, and then returned to their home cage. The water in the cylinder was renewed between subjects. Immobility behavioral scoring was performed by a rater who was blinded to the animal groups. Immobility was defined as behavior consisting of floating or movement of hind limbs directed exclusively to maintaining the head above water.

### Locomotor Activity

Locomotor activity has been used to evaluate depressive-like and anxiety-like behaviors (Kelly et al., 1998). In this test, each rat was placed into an open black wooden box (100 × 100 × 40 cm) that was enclosed in a soundproof box. A video camera connected to a computer was located over the box. Each rat was placed individually in this empty, novel arena and allowed to explore freely for 60 min. The total horizontal distance was automatically scored by a video tracking system (SMART video tracking system, MoblieDatum, China). After each test in the above experiments, the apparatus was cleaned with 70% ethanol solution.

### Rat Brain Dissection

The day after the last behavioral test, all experimental animals were deeply anesthetized with sodium pentobarbital (50 mg/kg, i.p.), and the PFC was isolated by using the method as previously described (Spijker, 2011). In brief, the skull was carefully opened to access the whole brain, using curved forceps to take out the whole brain and making sure that no mechanical damage was done. The entire PFC section was dissected, put in a 1.5 ml Eppendorf tube, snap-frozen with liquid nitrogen, then stored in a −80°C freezer.

### Western Blot Assay

Western blots were carried out by standard methods (Notaras et al., 2019). In brief, the PFC tissues were homogenized in ice-cold lysis buffer (10 mM Tris, 150 mM NaCl, 1% Triton X-100, 0.5% NP-40 and 1 mM EDTA at pH 7.4) containing a 1:100 (v/v) ratio of protease and phosphatase inhibitor cocktail (Roche). The protein samples were quantified using a Bicinchoninic Acid Protein Assay kit (Beyotime Institute of Biotechnology, Shanghai, China). Protein aliquots were mixed with an equal volume of loading buffer, boiled for 5 min, and loaded onto 12% gels before being subjected to SDS-PAGE. Subsequently, proteins in the gels were transferred to polyvinylidene difluoride (PVDF) membranes (Roche), incubated in 5% milk diluted in TBST for 3 h, and then incubated with primary antibodies including pAKT antibody (1:500; #9271s, Cell Signaling, MA, USA), AKT antibody (1:1000; #9272, Cell Signaling, MA, USA), CREB antibody (1:1000; ab32515, Abcam, Cambridge, UK), pCREB antibody (1:500; ab32096, Abcam, Cambridge, UK), pERK antibody (1:500; #4370s, Cell Signaling, MA, USA), ERK antibody (1:1000; #4695s, Cell Signaling, MA, USA), BDNF antibody (1:500; ab6201, Abcam, Cambridge, UK), and anti-β-actin antibody (1:1000; #4967, Cell signaling, MA, USA) at 4°C overnight. Membranes were washed in TBST (3 × 5 min) and incubated in corresponding secondary antibodies for 3 h at RT. Specific bands were detected and quantified by using a fluorescence scanner (Odyssey P140-CLx Infrared Imaging System, LI-COR Biotechnology, Lincoln, NE, USA).

### Statistics

All statistical analyses were performed in Prism v.8.0 (GraphPad Software, Inc. San Diego, CA, USA) and SPSS v.22.0 (SPSS Inc., Chicago, IL, USA). The results were expressed as mean ± SEM. One-way analysis of variance (ANOVA) and repeated-measures ANOVA followed by Tukey *post hoc* test was used for multiple comparisons. Figures were plotted using GraphPad Prism 8.0. Statistical significance was accepted when P < 0.05.

## Results

### Effects of AST on Body Weight, Fasting Plasma Glucose, and Glucose Tolerance

**Figure 2:**
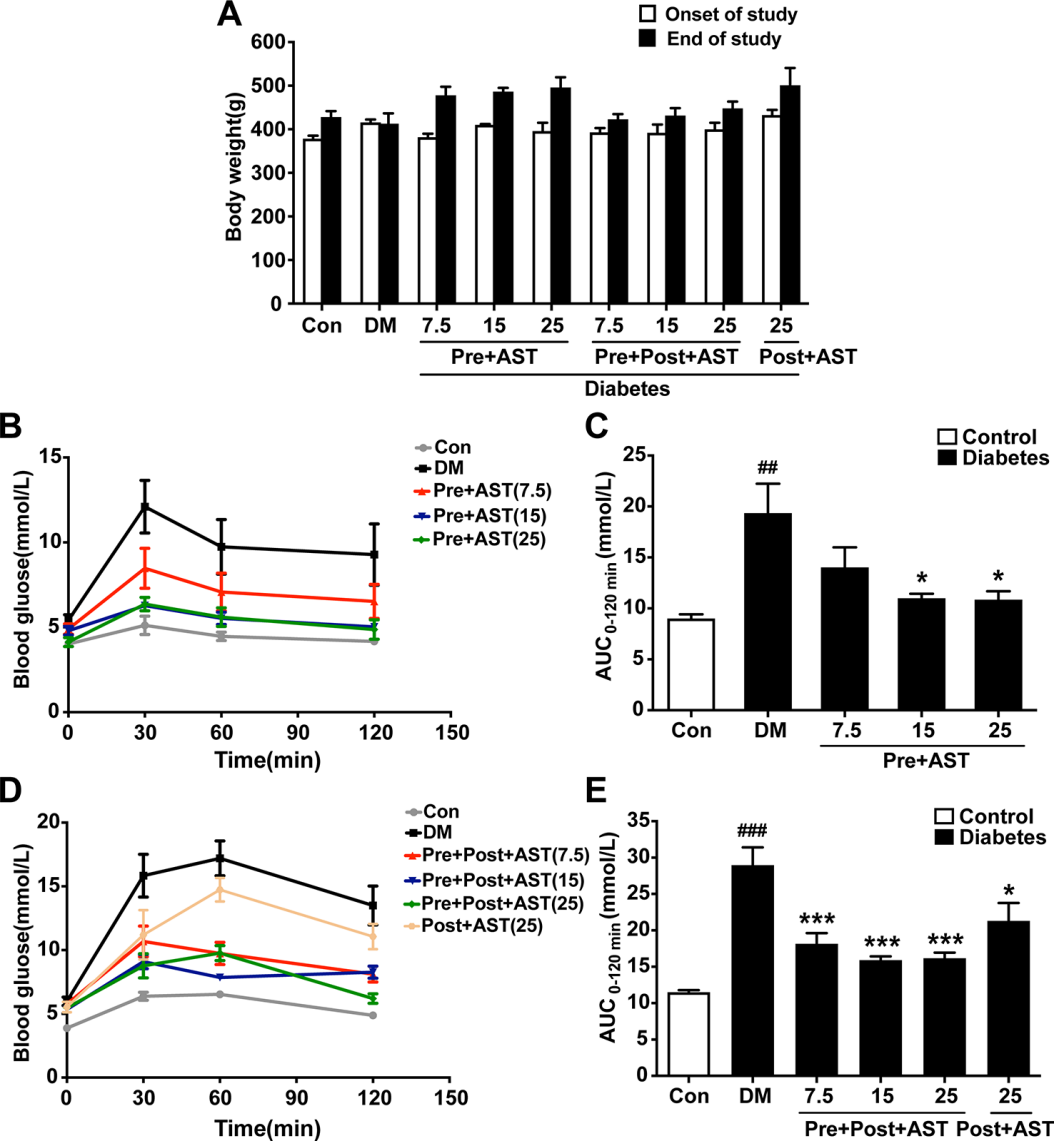
Effects of AST treatment on body weight, blood glucose level, and area under the curve of OGTT. Diabetic rats given AST showed no significant difference among groups in body weight **(A)** but improved glucose tolerance as measured by blood glucose **(B**, **D)** and area under the curve of OGTT **(C**, **E)** compared with diabetic vehicle rats. Data are shown as the mean ± SEM (n = 6). ^##^p < 0.01, or ^###^p < 0.001 vs. control group, ^*^p < 0.05, or ^***^p < 0.001 vs. DM group.

Repeated-measures ANOVA proved that there was no significant difference in body weight among the groups (F = 1.73, P = 0.41), but a trend was revealed that, with the AST treatment, rats would increase their weight (**Figure 2A**). As shown in **Figure 2B**, there were significant effects between the different groups (F = 14.87, P = 0.03) in terms of non-fasting plasma glucose at 30 min, 60 min, and 120 min one week after STZ injection. In the OGTT results represented as glucose AUCs, Pre+AST groups at doses of 15 and 25 mg/kg had significantly decreased glucose tolerance compared to the DM group (F = 6.30, p = 0.003, **Figure 2C**). As shown in **Figure 2D**, there was also a significant difference between the different groups (F = 10.19, P = 0.026) in terms of non-fasting plasma glucose at 30 min, 60 min, and 120 min at the end of the study. In the OGTT results represented as glucose AUCs, all of the AST-treated groups show a remarkable decrease in glucose tolerance compared to the DM group (F = 17.16, P < 0.001, **Figure 2E**). This finding implied that AST had no obvious effect on the body weight but significantly improved the glucose tolerance of the diabetic rats in this study.

### Effect of AST on Lipid Parameters in Plasma

**Figure 3:**
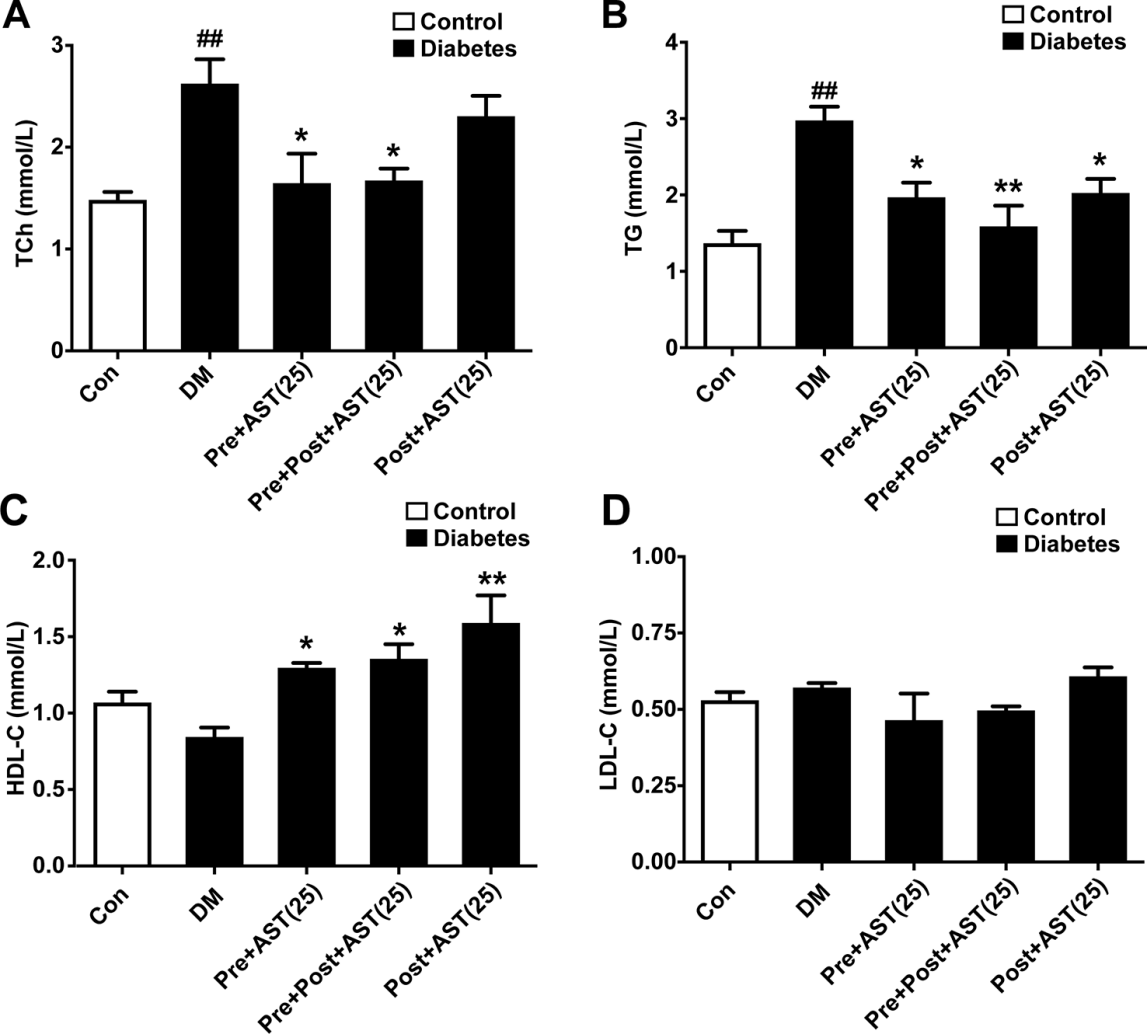
Effects of AST treatment on levels of blood lipids. AST reduced total cholesterol (TC) **(A)** and triglycerides (TG) **(B)** and increased high-density lipoprotein cholesterol (HDL-C) **(C)** but did not significantly change low-density lipoprotein cholesterol (LDL-C) **(D)** in diabetic rats. Data are shown as the mean ± SEM (n = 6). ^##^p < 0.01 vs. control group, ^*^p < 0.05, ^**^p < 0.01 vs. DM group.

One-way ANOVA revealed significant differences between the different groups in terms of TCh (F = 7.40, P = 0.001, **Figure 3A**), TG (F = 7.24, P = 0.001, **Figure 3B**), and HDL-C (F = 8.35, P = 0.002, **Figure 3C**). Briefly, the DM group had significantly increased TCh (P < 0.01) and TG (P < 0.01) and slightly reduced HDL-C (P > 0.05). In contrast, the AST-treated group (25 mg/kg) showed significantly decreased TCh (P < 0.05) and TG (P < 0.05) and significantly increased HDL-C (P < 0.05) compared with the DM group but no difference from the Post-treated AST group in terms of TCh (P > 0.05). As shown in **Figure 3D**, there was no significant difference between any of the AST treatment groups and the DM group in terms of LDL-C (F = 2.05, P = 0.13). These data indicate that AST effectively improved the lipid parameters in plasma in diabetic rats.

### Effects of AST on Depressive-Like Behaviors

**Figure 4:**
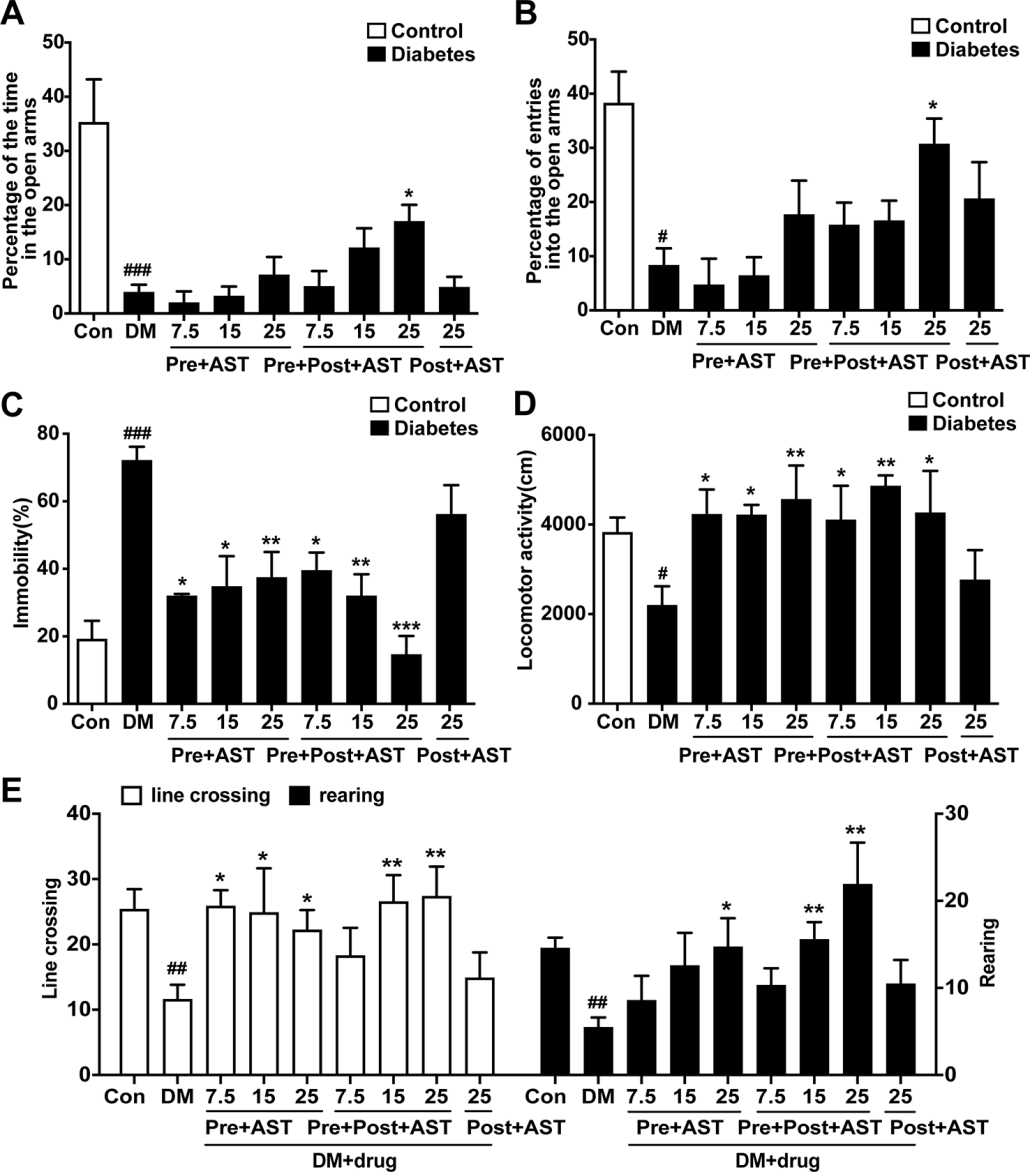
Effects of AST treatment on depression-like behaviors. This figure shows that AST could ameliorate the depressive-like behaviors of diabetic rats. Preventive plus therapeutic treatment with AST increased the time and the number of entries in the open arms of the elevated plus maze test **(A**, **B)**. Preventive or preventive plus therapeutic treatment with AST could decease the duration of immobility in the forced swimming test **(C)** and increase the locomotor activity of diabetic rats **(D)** and the frequency of line crossing and rearing in the open field test **(E)**. Data are shown as the mean ± SEM (n = 6). ^#^p < 0.05, ^##^p < 0.01, or ^###^p < 0.001 vs. control group, ^*^p < 0.05, ^**^p < 0.01, or ^***^p < 0.001 vs. DM group.

The effects of AST on the depressive-like behaviors induced in diabetic rats were examined at the end of the experiment. In the EPM, the time spent in the open arms decreased in the DM group (F = 8.48, P = 0.004, **Figure 4B**) and the percentage of entries into the open arms also decreased (F = 3.51, P < 0.001, **Figure 4A**). Only the Pre+Post+AST group at a dose of 25 mg/kg showed a significant increase in the time spent in the open arms (P < 0.05) and percentage of entries into the open arms (P < 0.05). As shown in **Figure 4C**, the FST results showed that AST treatment had a significant effect on the duration of immobility in diabetic rats (F = 9.07, P < 0.001), and multiple comparisons showed that the immobility time increased significantly in the DM group, while the Pre+AST and Pre+Post+AST groups at doses of 7.5, 15, or 25 mg/kg had a significantly decreased immobility time (P < 0.05). In contrast, Post-AST treatment after STZ injection failed to affect the immobility time (P > 0.05). Additionally, a significant increase was observed in the locomotor activity with AST treatment as measured by the open field test [(line crossing (F = 2.63, P = 0.02), rearing (F = 3.56, P = 0.004), as shown in **Figure 4E**, and locomotor activity (F = 2.23, P = 0.048), as shown in **Figure 4D**)]. The locomotor activity decreased in diabetic rats compared with control rats, and preventive or preventive plus therapeutic treatment with AST increased the locomotor activity in the diabetic rats. However, therapeutic treatment with AST alone did not influence the locomotor activity of the diabetic rats. These data revealed that AST could ameliorate the depressive-like behaviors of diabetic rats to a certain extent.

### Effects of AST on the Expression of pERK, pCREB, pAKT, and BDNF in the PFC

**Figure 5:**
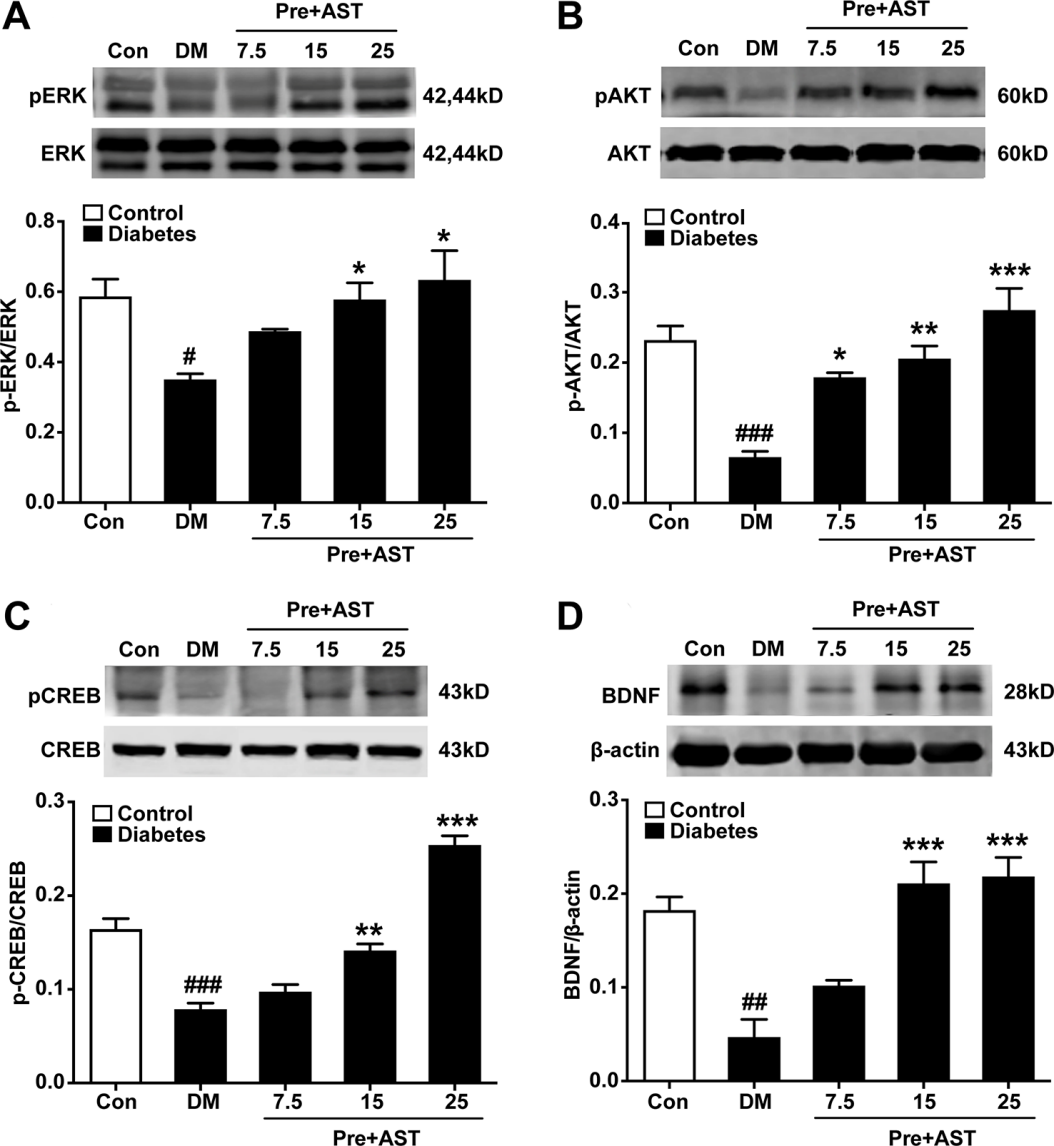
Effects of preventive treatment with AST on the relative expression levels of pERK1/2, pAKT, pCREB, and BDNF in the PFC. The representative bands of phosphorylated ERK1/2 **(A)**, phosphorylated AKT **(B)**, phosphorylated CREB **(C)**, and relative-expression BDNF **(D)** in the PFC and their histograms are shown. Data are shown as the mean ± SEM (n = 6). ^#^p < 0.05, ^##^p < 0.01, or ^###^p < 0.001 vs. control group, ^*^p < 0.05, ^**^p < 0.01, or ^***^p < 0.001 vs. DM group.

**Figure 6:**
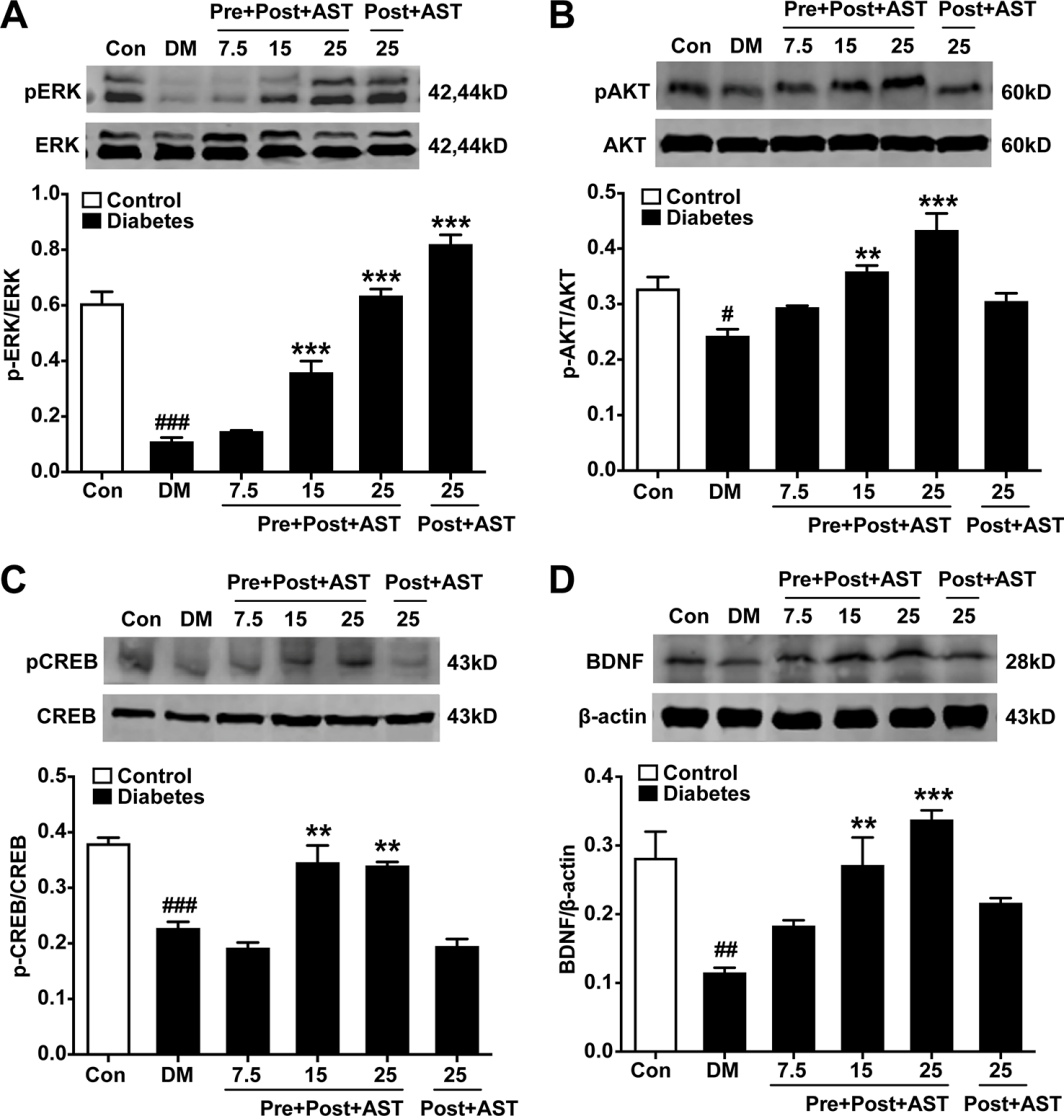
Effects of preventive or preventive plus therapeutic treatment with AST on the relative expression levels of pERK1/2, pAKT, pCREB, and BDNF in the PFC. The representative bands of phosphorylated ERK1/2 **(A)**, phosphorylated AKT **(B)**, phosphorylated CREB **(C)**, and relative-expression BDNF **(D)** in the PFC and their histograms are shown. ^#^p < 0.05, ^##^p < 0.01, or ^###^p < 0.001 vs. control group; ^**^p < 0.01 or ^***^p < 0.001 vs. DM group.

The preventive effects of AST on the relative expression of pERK, pAKT, pCREB, and BDNF in the PFC were examined at the end of the experiment. As shown in **Figure 5**, there were significant main effects of AST preventive treatment in the expression of pERK (F = 5.29, P = 0.015, **Figure 5A**), pAKT (F = 17.48, P < 0.001, **Figure 5B**), pCREB (F = 63.80, P < 0.001, **Figure 5C**), and BDNF (F = 18.50, P < 0.001, **Figure 5D**) in the diabetic rats. Expression of these proteins in the PFC decreased in the diabetic rats compared with control rats, and preventive treatment with AST at doses of 15 or 25 mg/kg significantly increased the expression of pERK, pAKT, pCREB, and BDNF in the PFC, respectively. As for detecting the therapeutic effects of AST, as shown in **Figure 6**, the statistics revealed that the expression levels of pERK (F = 97.39, P < 0.001, **Figure 6A**), pAKT (F = 14.45, P < 0.001, **Figure 6B**), pCREB (F = 30.27, P < 0.001, **Figure 6C**), and BDNF (F = 11.28, P < 0.001, **Figure 6D**) in the PFC were significantly different between the different groups. In brief, the expression levels of these proteins in the PFC of the diabetic rats were decreased compared to the control group (P < 0.05). Preventive plus therapeutic treatment with AST at doses of 15 or 25 mg/kg significantly increased the expression of pERK, pAKT, pCREB, and BDNF in the PFC, respectively, while therapeutic treatment with AST at a dose of 25 mg/kg only increased the expression of pERK but had no effect on the expression levels of pAKT, pCREB, and BDNF in the PFC.

## Discussion

The findings of the present study are that chronic preventive or therapeutic treatment with AST decreased the hyperglucemia and hyperlipemia induced by HFD and a low-dose STZ in a dose-dependent manner. Meanwhile, preventive treatment or preventive treatment plus therapeutic treatment with AST, followed by 6 weeks fed of HFD, improved the glucose tolerance and decreased the depressive-like behaviors induced by HFD and damage of islet cells, indicating that there was a positive relationship between the HFD and the development of depression and that chronic treatment with AST may improve the depressive-like behaviors. Moreover, preventive or preventive plus therapeutic treatment with AST increased the expression of pERK, pCREB or pAKT, and BDNF in the PFC, which correlated with the behavioral improvement with AST treatment in the diabetic rats. The data suggested that AST may be a potential agent for preventing diabetes and co-morbidity with depression induced by an HFD and the administration of a single dose of STZ.

Here, HFD feeding combined with damage of islet cells resulted in diabetes, which presented through characteristics such as increased fasting plasma glucose, impaired OGTT, and hyperlipemia (Luo et al., 2016; Ye et al., 2017). Long-term HFD feeding is known to produce systemic, chronic inflammation in animals and humans (Gregor and Hotamisligil, 2011; Buckman et al., 2014). Even after one day of HFD feeding, mice and rats show increased hypothalamic IL-1β and IL-6 levels, even before substantial weight gain could be detected (Thaler et al., 2012). Some studies show that an increase in fat mass enhances the secretion of pro-inflammatory cytokines such as TNF-α and IL-6 from adipocytes (Ouchi et al., 2012). The present data showed that AST, in a dose-dependent manner, improved the impaired OGTT and increased the HDL, which was consistent with reports that AST protects against inflammation (Ying et al., 2015) and attenuates oxidative stress (Li et al., 2016). AST is a carotenoid pigment with multiple pharmacological properties (Wu et al., 2015), and has a potent anti-inflammatory function (Grimmig et al., 2017). Thus, its anti-oxidative and anti-inflammatory effects may contribute to the preventive effect of AST on diabetes and depressive-like behaviors.

A combination of HFD feeding and STZ injection can successfully induce a rat model of diabetes (Srinivasan et al., 2005). The diabetic rats here exhibited a variety of depressive-like behaviors such as a reduction in the number of crossings and locomotion activity, a decrease in the time spent in the open arms, and an increase in immobility in the forced swimming test. We also used the sucrose preference test to assess anhedonia. There was no significant difference between the control and diabetes groups (shown in the **Supplementery Material**). It is possible that diabetic rats may be vulnerable to palatable foods, increasing food-motivated behavior (Sharma and Fulton, 2013). Thus, further research such as a saccharin choice test is warranted in diabetic rats. The changes in spontaneous activity in the diabetic rats could affect the interpretation of the results of EPM and FST. In fact, whatever the percentage of time spent in the open arms as well as of entries into the open arms in EPM and the immobility time in the FST, these changes in amplitude were greater than that of spontaneous activity in the diabetic rats. This implied that alteration of the time spent in the open arms and in immobility time was not entirely due to a reduction in locomotion activity.

Epidemiological data indicate that individuals with obesity have an increased risk of developing mood disorders such as major depressive disorder (Mansur et al., 2015). Clinical data have shown that obesity is a risk factor for depression disorders (Luppino et al., 2010). Chronic inflammation caused by an HFD plays a major role in inducing depression (Sharma and Fulton, 2013; Dutheil et al., 2016). The present data showed that preventive or preventive plus therapeutic treatment with AST could improve both glucose and lipid metabolism and consequently decrease depressive-like behaviors. Moreover, preventive plus therapeutic treatment with AST displayed more beneficial effects on the dysfunction of metabolism and depressive-like behaviors. Meanwhile, this means that islet damage due to STZ injection may not only worsen the glucose metabolism function but also induce depressive-like behaviors synergistically with HFD feeding. Interestingly, therapeutic treatment with AST at the maximal dose failed to affect depressive-like behaviors, although it did improve glucose tolerance and fasting glucose level, implying that HFD feeding might be a causative factor for the pathogenesis and development of depression.

BDNF has been widely related to the pathogenesis of depression disorders. A decreased level of BDNF in the hippocampus, PFC, or serum correlates with depression in animals and humans (Bocchio-Chiavetto et al., 2010; Autry and Monteggia, 2012). HFD feeding attenuates the hippocampal BDNF level in rodents (Molteni et al., 2002). On the other hand, an elevated BDNF level in the nucleus accumbens and ventral tegmental area of HFD-fed mice results in depressive-like behaviors (Sharma and Fulton, 2013). BDNF and its receptor, tropomyosin-related kinase B (TrkB), are potential therapeutic targets for major depressive disorder (Zhang et al., 2014). The present results showed that a decrease in the BDNF level in the PFC was observed, and pretreatment with AST reversed the reduction of BDNF in this diabetes co-morbidity with depression model. A few studies demonstrate that AST decreases depressive-like behaviors induced by LPS or in diabetic mice *via* its potent anti-inflammatory property (Jiang et al., 2016; Zhou et al., 2017). Thus, the increased BDNF level in the PFC following treatment with AST indicated that neuroplasticity regulation by AST is crucial for its antidepressant activity.

Reductions in the BDNF level in the PFC induced by HFD and diabetes are implicated in the production of depression, which is achieved by activation of CREB (Yu and Chen, 2011). After binding to its receptor, TrkB, BDNF initiates both the phosphatidylinositol 3-kinase (PI3K)/AKT and mitogen activated protein kinase (MAPK)/ERK pathways, which then elicits gene transcription and translation through CREB factor (Marsden, 2013). These BDNF-regulated signaling cascades are responsible for synaptic plasticity, spine morphogenesis, and neurogenesis (Park and Poo, 2013). Chronic treatment with AST inhibits acetaldehyde-induced decrease in activated AKT and CREB levels (Yan et al., 2016). Evidence has also shown that AST activates the PI3K/AKT signaling pathway (Wang et al., 2016). The present results showed a reduction in the phosphorylation of CREB, ERK1/2, and AKT in the PFC in the diabetic rats. Meanwhile, the preventive or preventive plus therapeutic treatment with AST could reverse the decreased pCREB, pERK and pAKT in diabetic rats. AST treatment, whether before or after STZ injection, could improve the glucose tolerance, but only preventive treatment or preventive plus therapeutic treatment with AST could improve the depressive-like behaviors, which correlated with the up-regulation of BDNF and its signaling pathway in the PFC. Therefore, the up-regulation of BDNF and its signaling pathways in the PFC may be involved in the antidepressant effect of AST in diabetes.

The relationship between obesity and depression is bi-directional. Despite the introduction of new antidepressants, many obese patients with comorbid depression respond poorly to therapy, suggesting that obesity may diminish the efficacy of antidepressant treatment (Woo et al., 2016). Recent studies have shown that combination therapies increase the levels of insulin and monoamines, reduce oxidative stress and production of proinflammatory cytokines, and can potentially be used to treat type 2 diabetes mellitus and co-morbid depression (Shivavedi et al., 2017). Accumulating evidence suggests that the mechanism of AST in preventing depression or diabetes may involve the inhibition of inflammation, thereby protecting neurons in the PFC against hyperlipemia and hyperglycemic damage. Moreover, the expression of BDNF and its signaling pathway in the prefrontal cortex is significantly decreased, which is helpful for understanding the pathogenesis of diabetes co-morbid with depression. Chronic treatment with AST had a preventive or therapeutic beneficial effect for metabolism syndrome and depression.

## Conclusions

This study revealed that co-morbidity of diabetes and depression was established by feeding rats with an HFD and a low dose of STZ treatment. The results demonstrated that AST pretreatment, whether before or after STZ injection, could improve glucose tolerance but that only preventive or preventive plus therapeutic treatment with AST could improve depressive-like behaviors, which correlated with the up-regulation of BDNF and its signaling pathway in the PFC. The data suggested that AST may be used as in preventive and therapeutic approaches for co-morbid diabetes and depression disorders.

## Data Availability Statement

The datasets generated for this study are available on request to the corresponding authors.

## Ethics Statement

The experimental protocols were approved by Institutional Animal Care and Use Committee of Ningbo University.

## Author Contributions

YK, SB, YZ, and WZ conceived the experiment, YK, HM, LG, and YC conducted the experiments, and YK, SB, and WZ analyzed the results. All of the authors reviewed the manuscript.

## Funding

This work was supported by the Natural Science Foundation of China (81671321), the Natural Science Foundation of Ningbo (2019C50091), and the National Basic Research Program of China (2015CB553504).

## Conflict of Interest

The authors declare that the research was conducted in the absence of any commercial or financial relationships that could be construed as a potential conflict of interest.
