# Supplementary material for: Preventive and Therapeutic Effects of Astaxanthin on Depressive-Like Behaviors in High-Fat Diet and Streptozotocin-Treated Rats
Source: Front Pharmacol. 2020 Jan 30;10:1621. doi: 10.3389/fphar.2019.01621 (PMC7003134; doi:10.3389/fphar.2019.01621)
Supplement: Supplementary file 1 [file DataSheet_1.doc]

**Supplementary Material**

**Effect of AST treatment on the sucrose preference in the diabetic rats**

**METHOD**

**Sucrose preference test**

The sucrose preference test has been used extensively to assess anhedonia, through changes in sucrose consumption. This test was performed as described earlier(Willner, Towell, Sampson, Sophokleous, & Muscat, 1987) with minor modifications. Briefly, 72h before the test, rats were housed in individual cages to acclimate with two bottles of 1% sucrose solution (w/v) for 24 h, and one of the sucrose bottles was replaced with tap water for the next 24h. After the adaptation, rats were deprived of water and food for 24 h, followed by the sucrose preference test, in which rats had free to access to two bottles with one containing 400 ml of 1% sucrose solution (w/v) and the other containing 400 ml of tap water over a 24-h period. The positions of the two bottles were switched after 12 h of testing to prevent possible effects of side preference. For each group, the consumption of sucrose solution and water was measured by weighing the bottles. The sucrose consumed was calculated by the following formula: sucrose preference (%) =(sucrose consumption) / (sucrose consumption +water consumption)×100%.

**RESULT and DISCUSSION**

The one-way ANOVA revealed that there was no significant difference between control and diabetes groups (F =1.11, P=0.37, Figure S1) in sucrose consumption in the sucrose preference test, but there was a trend exhibited that Preventive or Preventive plus therapeutic treatment with AST at the dose of 25 mg/kg slight an increase in sucrose preference compared with DM group. A few studies reported that HFD fed or STZ-induced diabetic animals shown anhedonia in a sucrose preference test (Kurhe, Mahesh, & Gupta, 2014; Tang et al., 2019), however, in this study, there was no significant difference between control and diabetes groups. It is possible that the diabetic rats might be vulnerability for palatable foods by increasing food-motivated behavior (Sharma, Fernandes, & Fulton, 2013). Thus, further research such as saccharin choice test is warranted in the diabetic rats.

**
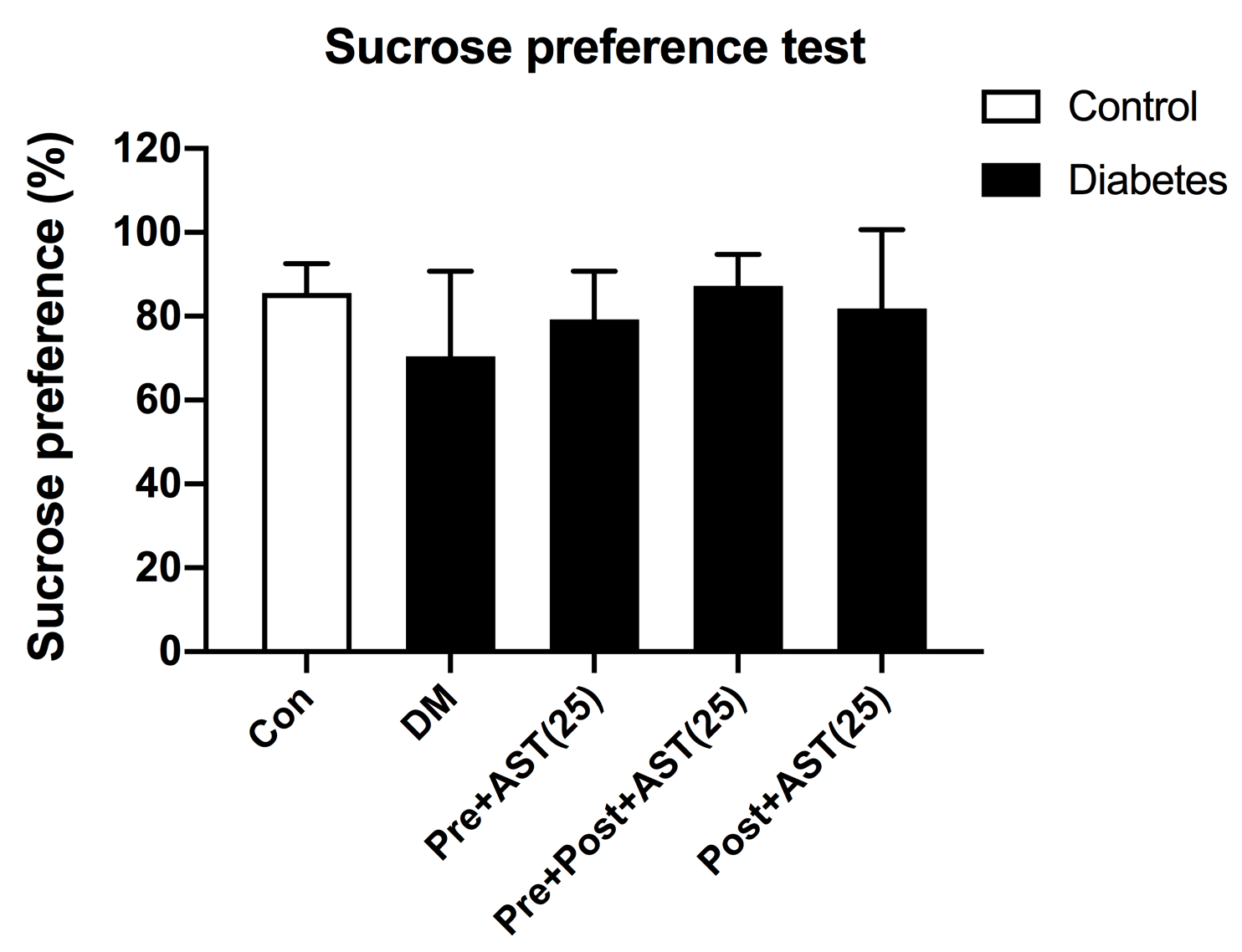
**

**Figure S1. Effect of AST treatment on the** **sucrose preference in the diabetic rats.** The data is presented as mean±SEM (n=6). Con: normal animal; DM, diabetic animals; Pre+AST, preventive treatment with AST; Pre+Post+AST, preventive plus therapeutic treatment with AST; Post+AST, therapeutic treatment with AST; AST(25), astaxanthin (25mg/kg). This figure revealed that after 24h of sucrose consumption, there was no significant difference between each group (F =1.11, P=0.37).

**REFERENCE**

Kurhe, Y., Mahesh, R., & Gupta, D. (2014). Effect of a selective cyclooxygenase type 2 inhibitor celecoxib on depression associated with obesity in mice: an approach using behavioral tests. *Neurochem Res, 39*(7), 1395-1402. doi: 10.1007/s11064-014-1322-2

Sharma, S., Fernandes, M. F., & Fulton, S. (2013). Adaptations in brain reward circuitry underlie palatable food cravings and anxiety induced by high-fat diet withdrawal. *Int J Obes (Lond), 37*(9), 1183-1191. doi: 10.1038/ijo.2012.197

Tang, C. R., Yu, X. B., Zhang, H. N., Cao, Y. C., Yang, F., Xu, L. M., . . . Liang, J. (2019). Lovastatin Prevents Depressive Behaviors and Increased Hippocampal Neurogenesis in Streptozotocin-Induced Diabetic Mice. *Pharmacology*, 1-10. doi: 10.1159/000503865

Willner, P., Towell, A., Sampson, D., Sophokleous, S., & Muscat, R. (1987). Reduction of sucrose preference by chronic unpredictable mild stress, and its restoration by a tricyclic antidepressant. *Psychopharmacology (Berl), 93*(3), 358-364. doi: 10.1007/bf00187257
